# A Qualitative Study of Nursing Students' Experiences in Fall Prevention for Older Home Care Clients

**DOI:** 10.1155/2020/7652623

**Published:** 2020-06-28

**Authors:** Riitta Turjamaa, Marja Äijö, Tarja Tervo-Heikkinen, Marja Silén-Lipponen

**Affiliations:** ^1^Savonia University of Applied Sciences, School of Health Care, Kuopio, Finland; ^2^University of Eastern Finland, Kuopio, Finland; ^3^Clinical Education and Research Unit of Nursing, Kuopio University Hospital, Kuopio, Finland

## Abstract

The aim of this study was to describe the experiences of nursing students in fall prevention during clinical practice in the context of older home care clients. This was a qualitative focus group study of nursing students (*n* = 9) who had completed clinical practice in older clients' home care. The data were analysed using inductive content analysis. The nursing students described their experiences regarding falls and fall prevention in older clients' home care from two perspectives: evaluation of falls at older people's homes and fall prevention during home visits. Systematic evaluation of falls was based on physical examination and is the basis of fall prevention. However, evaluation of nutrition and adverse drug effects seemed to be ignored. In addition, fall prevention during home visits included concrete fall prevention in authentic client situations, confidential relationships with older clients, and evidence-based knowledge. From the perspective of fall prevention, there was a lack of comprehensive evaluation and understanding of the meaning of psychological factors, such as fear of falling. In order to be able to prevent falls in the older client population, students need more guidance regarding a comprehensive approach based on evaluation of falls. In addition, there is a need for continuous collaboration between education and home care services to develop educational approaches that interlink knowledge and skills in fall prevention.

## 1. Introduction

The majority of older people are healthy and live at home in familiar environments [1]. However, as older people become even older, the risk of multiple diseases and decreased functional ability can increase the risk of falls and fall-related injuries [2–4]. According to the World Health Organization [5], older people's falls are described as “inadvertently coming to rest on the ground, floor, or other lower levels, excluding any intentional change in position to rest on furniture, walls, or other objects.”

Fall prevention is a significant part of supporting older people's independent living [6, 7], and it is therefore globally an important area of development in the field of healthcare. It is estimated that more than one-third of people aged 65 or older experience falls [8], causing suffering, functional decline [9], and increased need for healthcare services that incur significant financial costs for healthcare [10].

Based on earlier studies, several risk factors for falls and their consequences among older people have been identified [6, 11]. The main situations leading to falls are walking and moving, especially going to the bathroom at night [12]. In addition, previous falls, age, balance impairments, decreased cognition, cardiovascular diseases [11], and medications [6] are the most significant risk factors for falls among older people living in their own homes. Furthermore, it has been recognised in previous studies that there is a connection between falling and fear of falling [4, 12]. Falls at home can also lead to long-term institutional care and even death [13]. Moreover, it was found that older people have a higher risk of death if they had fallen two or more times [6].

Risk factors for falls can be classified as intrinsic or extrinsic and environmental factors [3, 14]. Intrinsic factors are related to the person himself/herself, such as diseases, lower extremity weakness, balance disorders, functional and cognitive impairments, and visual deficits. Extrinsic factors include, for example, polypharmacy and the use of certain medications, as well as environmental factors at home, including poor lighting, slippery floors—especially in bathrooms—and small rooms with ample furniture that partly obstructs walkways [11, 15]. On the contrary, the connection between risks for falls at home and older people's ability to function in their environment is complex, and therefore, a simple cause-and-effect relationship cannot be established [16]. However, older people who have fallen or are at high risk for falls should be recognised and evaluated. A few falls risk-assessment tools for older people living at home have been developed and validated, such as the Falls Risk for Older People in the Community (FROP-Com) setting assessment tool [17] and the Falls Risk Assessment Tool (FRAT) [18]. In addition, the Falls Efficacy Scale International (FES-I) [19] was developed to evaluate fear of falling in older people.

Home care professionals can play a significant role in recognising and evaluating risks for falls during home visits. However, based on previous studies, it can be a challenge for professionals to take into account risks of falls [6, 13, 16] and to support older home care clients in understanding and recognising their risks of falls in home surroundings [16]. In addition, nursing students will be in the key role in the future of preventing older clients' falls when working as professionals. However, our preliminary search of the literature showed that research on nursing students' experiences in relation to older clients' falls and fall prevention is still lacking, and further research is needed. Therefore, the aim of this study was to describe the experiences of nursing students in fall prevention during clinical practice in the context of older clients' home care.

## 2. Materials and Methods

### 2.1. Study Design

This was a qualitative focus group study of nursing students who had completed clinical practice in older people's home care. Focus group interviews were chosen because participants can talk to each other and discuss their answers, generating data about their experiences and observations related to falls and fall prevention, which can be a challenging issue for students. Therefore, this was a useful method for finding out about shared and individual experiences [20].

This study was a part of the AKESO study coordinated by Savonia University of Applied Sciences (SUAS), which was a development and research project. The aim of the AKESO study [21] was to develop effective teaching methods to support nursing students learning about fall prevention among older people. SUAS is in alliance with the Regional Fall Prevention Network [22] that operates in the region of the Kuopio University Hospital (KUH) district. The aim of the collaboration is to produce new information for educational development to ensure that students are given enough evidence-based knowledge and practical experiences about how to prevent falls. As a whole, the AKESO study consisted of three data collection phases: (1) during the beginning of a gerontological nursing course; (2) during an authentic simulation situation at SUAS; and (3) after clinical practice in the context of older clients' home care.

### 2.2. Participants and Data Collection

Participants were nursing students (*n* = 9) who had experience working in home care services with home care clients. The mean age of the participants was 23 years, and their mean length of study in nursing was three years. The participants met the following criteria: (1) voluntary participation in the study; (2) completion of a gerontological nursing theoretical course; and (3) clinical practice experience as a nursing student in the context of older client home care.

One author, also being the teacher of the student group during their gerontological nursing practical placement period, send the e-mail to the student group. The students were informed about the aims of the study, conducting interviews, and the voluntary and confidential nature of their participation. A total of 23 students received the e-mail, and nine students expressed willingness to participate in the study. After that, another author of this manuscript who did not know the students before and was not their teacher contacted participating students and organized and conducted the interviews. Interviews were conducted in a meeting room at the University Campus during May 2017. Two focus groups, each with four and five participants (*n* = 9), were conducted by the same researcher. Themes used in the interviews were (1) evaluation and (2) prevention of falls in the context of older people's home care. Focus groups lasted from 40 to 45 minutes.

### 2.3. Data Analysis

Data were analysed using inductive content analysis [23]. The recorded data were transcribed by one researcher and consisted of 19 A4 pages with 1.5-line spacing. The first step was to read through all of the material to get an overview of the interviews. Second, single words and combinations of words or sentences were identified from the data (e.g., “Homes should be roomier. Not so many things on the floor”). Identified meaning units were grouped into subcategories (e.g., messy home), and subcategories were again abstracted to create main categories (e.g., systematic evaluation of falls) from which overall themes were created (e.g., evaluation of falls at an older client's home) [23]. The analysis was conducted by two researchers.

### 2.4. Ethical Considerations

Based on legislation [24, 25], the Research Ethics Committee has to evaluate the research plan in case of need, for example, when vulnerable groups are involved in a study. In this study, the participants were adult students, and thus, we did not need the evaluation of the Research Ethics Committee. The researchers obtained permission from the University of Applied Sciences before conducting the research. All participants were informed, both orally and in writing, that participation was voluntary, and that they had the right to withdraw at any time. Participants were told that interview material was confidential and only those participants who were involved in the study can handle the material. After being provided with information about the study, participants had time to consider their participation. Students were not reminded or persuaded. Finally, written informed consent was obtained from all participants, all information was treated confidentially, and the data were kept in a researcher's computer, which was protected with a user name and password.

## 3. Results

The nursing students described their experiences with falls and fall prevention in older clients' home care from two perspectives: (1) evaluation of falls at older clients' homes and (2) fall prevention during home visits (Table 1).

### 3.1. Evaluation of Falls in Older People's Homes

Based on our results, evaluation of falls in older clients' homes consists of the evaluation of functional ability and systematic evaluation of falls supported by supervising nurses who are working in older people's home care as professionals. Fall prevention can be described as an illness-centred approach that focuses only on clients' physical disabilities and chronic illnesses. Their individual resources were not taken into account. In addition, evaluation of falls is hasty by nature and, as such, fragmented in practice. However, the confidential relationship between the older client and students and supervisors created a connection between them based on reciprocal confidence and enabled discussions about risk factors for falls. In addition, the use of evidence-based knowledge was emphasised as a significant basis for students' professional development.

#### 3.1.1. Evaluation of Functional Ability

The students assessed that the evaluation of the functional ability of an older client is an essential part of the evaluation. For example, acute medical illness, muscle weakness, or mobility related to pain should be evaluated. Therefore, vital signs, orthostatic blood pressure, and body weight should be obtained regularly during home visits.

They have many diseases and their ability to function has decreased. Because of that, they do not dare to move and they are scared of their ability to walk. I think that we should take into account in many different ways and reasons for falls, such as pain and blood pressure.

#### 3.1.2. Systematic Evaluation of Falls

The students thought that a systematic evaluation of falls was the basis for fall prevention. Furthermore, all students stated that the systematic evaluation of falls is based on knowledge of available assessment tools, such as screening tools and questionnaires. They mentioned that practicing the use of different screening tools and questionnaires at school confirmed their understanding of the meaning of evaluation of falls. They also said that exploring the different evaluation methods helped them to be ready to use these methods in different contexts with different clients.

In practice, students expressed that using the different evaluation methods, such as questionnaires, made them ready to use these methods in different contexts with different clients. However, the students recognised that nurses did not systematically use assessment tools to evaluate clients' risks of falling. Furthermore, students noted that nurses' use of assessment tools was mechanical, routine-like, and did not include a client-centred approach. They also thought that this kind of action may hide the reasons for falls, especially with familiar clients.

Did we have assessment papers with us… maybe? We did not have time to interpret different papers. Maybe nurses observe their client's home but they do not ask or evaluate risks for falls with clients.

Students emphasised that they evaluated reasons for falls in daily home visits, including observation of older clients' use of drugs, impaired balance, impaired physical ability to function, and a messy home. Drugs especially were singled out as a remarkably common reason for falls. They described drugs, such as sedatives, and the simultaneous use of blood pressure medication causing vertigo and therefore causing falls.

They have lot of drugs. It is important to take drugs on time. I think that it is quite easy to observe drugs which may cause falls.

### 3.2. Fall Prevention during Home Visits

#### 3.2.1. Concrete Fall Prevention in Authentic Client Situations

The students said that fall prevention during home visits was based on real-life fall prevention in authentic client situations. In home visits, despite the presence of professionals, the students emphasised their own responsibility for fall prevention. The authentic client situations were meaningful for their professional development in preventing falls.

It was exciting to go to the unfamiliar home. My supervisor allowed me to first watch and then participate. No hurry to get to know.

Moreover, they emphasised that practicing the use of different screening tools and questionnaires at school enabled them in authentic situations to increase their clinical competence by observing and evaluating a client's situation at home from the perspective of evidence-based knowledge. In practice, this enabled students to apply information about fall prevention, such as fall-related risk factors. In addition, students emphasised confidential discussions with supervisors as remarkable when recognising reasons for falls at home. This was particularly true for clients who were at a very high risk for falls, which led to thorough discussions of the client's fragile situation at home and different methods for fall prevention.

It was nice to discuss with my own supervisor and others, too. I think it was good to hear theoretical things and then I know what these things mean in practice. I value supervisors' knowledge highly. It is important to hear it.

In daily home visits, students focused on the older clients' surroundings, including the availability and use of personal aids and safe shoes. Students considered that it might be easy to evaluate older clients' homes because they could make evaluations by looking around and seeing the risky things that can cause falls.

Shoes have to be sturdy; no wool socks.

The personal aids help the client to move and support living at home.

#### 3.2.2. Confidential Relationship with Older Clients at the Multifaceted System

Students felt that confidential discussions with clients about risks of falls made possible the concrete fall preventions, although with some clients, it was challenging to justify their personal needs for fall prevention. However, students emphasised the importance of a confidential relationship between an older client and a student based on reciprocal confidence, a relationship in which the student recognizes and considers the older client's autonomy and respects the way clients live in their own homes. For example, students decided in collaboration with the client whether there was a need to move furniture or remove rugs.

Homes should be roomier. Not so many things on the floor. However, I cannot say to the unfamiliar clients that they have to reorganize their home. Not so easy, I think.

Despite the focus on concrete fall prevention, and even the illness-centred nature of evaluations, nursing students found that there should be more shared positive views of clients' conditions than manifested, in particular, in the resource-based approach. In practice, students noted that clients were seen as passive persons with reduced abilities, and thus, their needs and resources were ignored. It can be a challenge for both students and home care professionals to identify and consider clients' resources. The students also recognised that professionals were even from different organizations, such as private service providers or associations. From student's point of view, home visit looked like professionals had defined the tasks that they must perform and specified the amount of time in which professionals should conduct each task. In sum, the students highlighted the need for continuing follow-ups regarding risks of falls. This means that all risks that have been recognised should be evaluated and observed regularly, based on clients' individual needs and resources.

Sometimes, I was wondering that we always looked for negative things at clients' homes: you are using risky shoes, you have so many sedative drugs, and you have muscular weakness and so on. How about things, which make easier older people's living at home and their strengths? Maybe it is easier to recognize weaknesses because we can see what older people cannot do. On the contrary, I think that resources are subjective experiences of abilities and therefore, it is not so easy to recognize these aspects. However, it is no explanation; the resource-based approach should be authenticated.

In addition, students pondered that home care seems to focus on care provided of several services and employees. The students felt that the nature of home care was fragmented, and employee's turnover was high, and thus, employees were unfamiliar with their clients and students as their supervisors. In that sense, students described fall prevention as a multiprofessional activity, but fragmented referring to the many employees.

From time to time, I have difficulties to understand the nature of home care. Especially older people with memory disorders are confusing when new nurse comes to visit rushed working habits. That is true, because I find it hard as a young people to keep up with all nurses.

#### 3.2.3. Evidence-Based Knowledge

In addition to confidential relationships with older clients, evidence-based knowledge was found to be a meaningful basis for students' professional development. For example, observing a supervisor's strategy for observing and evaluating a client's situation at home with regard to information about fall prevention, such as fall-related risk factors, illustrated how to apply evidence in real practice. Moreover, students emphasised the importance of discussions with supervisors, especially regarding clients who were at a very high risk for falls. This situation led to thorough discussions with the supervisor about the client's fragile situation at home and different methods for fall prevention in the future.

During home visits, I repeated with my supervisor the main points of risk for falls which I had previously learned at school.

Overall, students agreed that supervisors gave them time to determine clients' situations, such as the nature of their diseases and medications. Moreover, supervisors encouraged the students to notice the client's current situation during home visits. However, these types of situations are time-consuming, requiring more time for conversations between students and supervisors. Therefore, students reported that despite the strong role of evidence-based knowledge as the subject matter, the approach was narrow because the actual evaluation and fall prevention efforts were evidently routine-like and included no individual evaluations or observations.

Quite often my supervisor said that we should do this and this, but there is no time. I think that supervisors have knowledge and skills, but time is so limited.

## 4. Discussion

### 4.1. Discussion of the Main Results

In this study, we found that nursing students described their experiences with fall prevention during clinical practice with older clients from different perspectives, focusing on evaluation of falls and concrete fall prevention strategies during home visits.

*Evaluation of falls at older clients' homes based on systematic evaluation*. Although observing the use of drugs was highlighted during home visits, monitoring the adverse effects seemed to be ignored. However, adverse effects are common among older people and are the causes of hospitalisation in this population [26]. Another important fact that emerged was insufficient observation of nutrition. None of the descriptions contained systematic evaluations of nutrition, including any attention paid to proper or sufficient nutrition or the possibility of malnutrition.

Systematic evaluation as a whole was reported as being an important aspect of fall prevention. All students described conducting systematic evaluations of falls related to their knowledge of available assessment tools, such as screening tools and questionnaires. Students mentioned that practicing the use of different screening tools and questionnaires at school confirmed their understanding of evaluation of falls in practice [27]. They also said that exploring the different evaluation methods equipped them to use these methods in different contexts with different clients. However, they reported that evaluation tools during home visits were missing; it was a major issue neglected by supervisors and, therefore, partly by students as well. Overall, based on previous studies, it can be a challenge for professionals to use screening tools and questionnaires [6, 13, 16] to support older people in understanding and recognising the risk of falls in home surroundings [16].

In older people's home care, multiprofessional collaboration has been reported as playing an important role in sharing collected information, decision-making, and action related to fall prevention. In addition, it is significant that older clients take an active part in multiprofessional collaboration. They are experts on their own lives, and each client brings his or her own expertise to the collaboration and fall prevention, which extends their lives [6, 7] and therefore is globally an important development area in the field of client-oriented home care.

However, from the nursing students' perspective, fall prevention was described as disunited care that consisted of several employees unknown to the client with unclear responsibilities and focusing on limited risk factors for falls. Our study showed that nursing students described home care professionals' roles as based on expert orientation in the multiprofessional group but with an illness-centred orientation and scattered duties of each individual employee unknown to the client [6, 13, 16], which has also been reported in previous studies. Nursing students considered that the evaluation of falls was not based on a systematic approach. First, the use of different screening tools and questionnaires was irregular. Second, evaluations did not focus on risk factors, and therefore, there was a risk of losing the holistic overview of a client's situation. However, based on earlier studies, several risk factors for falls and their consequences among older people have been identified [6, 11].

Our results are similar to those of others regarding the relationship between students and supervisors and students' perspectives on supervisors' clinical competence in relation to evidence-based knowledge [28, 29]. It is evident that students' supervisors need evidence-based knowledge about fall prevention even though it is important for students to be active seekers of knowledge to achieve a professional level of fall prevention. This requires that supervisors consider the importance of evidence-based knowledge and uniquely tailor their supervising to reflect the students' learning needs. Such supervising and positive relationships support students in developing the necessary nursing skills to become competent and knowledgeable professionals, which has also been reported in previous studies [30–32].

In sum, the results highlight nursing students' experiences of daily home care, which includes dealing with preventing older clients from falling in their homes. Some essential questions concern the factors that support older clients' fall prevention at home. As home care clients grow older, it is evident that the lack of systematic evaluations of falls is causing more falls and therefore increasing public health and financial concerns. Moreover, professional-based fall prevention, which ignores older clients' opinions, cannot respond to the challenge of promoting clients' living at home for as long as possible. Additionally, an illness-centred philosophy is not responsive to the challenge of promoting students' development of the necessary nursing skills that incorporate a more preventive approach.

### 4.2. Limitations of the Study

There are some limitations associated with this study. Our results do not represent all nursing students' learning experiences in fall prevention during clinical practice in the context of older client home care. Therefore, no generalized conclusions can be proposed. This is important to consider in evaluating the results. The method used was focus group interviews; however, it is possible that individual interviews have provided deeper insights into the topic. The reason for the use of focus groups as a data collection method was based on the fact that this approach can be successful in obtaining multidimensional descriptions and diverse views about the learning experiences of nursing students. In addition, one of the researchers had an existing relationship with some participants as their teacher. This may have caused some students to feel that they needed to participate in the research. Before the interviews, the participants had received evaluations for both a gerontological nursing theoretical course and clinical practice in older people's home care. Therefore, participants did not feel that they were obliged to participate in research to get a better grade. Moreover, the amount of rich data obtained shows that participants enjoyed talking about their experiences and bolsters confidence in the results.

## 5. Conclusions

The results of our study provide information regarding nursing students' experiences with regard to fall prevention during clinical practice in the context of older clients' home care. Students' descriptions focused on evaluations of falls and fall prevention during home visits. However, the students' perspective was limited to just the physical environment and physical abilities. There was a lack of understanding of the role of psychological factors, such as fear of falling. In order to prevent falls in older clients living at home, students need more guidance on a comprehensive approach to evaluation of falls. Therefore, there is a need for continuous collaboration between nursing education and home care services to develop content that interweaves knowledge and skills in fall prevention. The collaboration should be strengthened by offering shared continuing education to all participants in the context of gerontological nursing.

## Figures and Tables

**Table 1:** The nursing students' experiences for fall prevention during clinical practice in the context of older people home care.

Themes	Main categories	Subcategories
Evaluation of falls at older people's home	Comprehensive physical examination	Acute medical illness
		Muscle weakness
		Mobility related to pain
	Systematic evaluation of falls	Knowledge of available assessment methods
		Low use of assessment tools
		Use of drugs
		Impaired balance
		Messy home

Fall prevention during home visits	Concrete fall prevention in authentic client situations	Observe home's internal surroundings
		Observe home's external surroundings
		Look after the use of safe shoes
		Take care of sufficient availability of personal aids
	Confidential relationship with older client at the multifaceted system	Reciprocal confidence
		Respect of autonomy
		Multiprofessional, but fragmented care
	Evidence-based knowledge	Comprehensive knowledge of related factors on fall prevention
		Low use of evidence-based knowledge

## Data Availability

The qualitative data used to support the findings of this study are restricted by the Savonia University of Applied Sciences in order to protect research subject privacy. Data are available from Riitta Turjamaa at the Savonia University of Applied Sciences, Unit of Health Care, Microkatu Campus, 70201 Kuopio, Finland, for researchers who meet the criteria for access to confidential data.
